# Group A *Streptococcus dysgalactiae* subspecies *equisimilis* vertebral osteomyelitis accompanied by progressive atlantoaxial subluxation: A case report and literature review

**DOI:** 10.1097/MD.0000000000034968

**Published:** 2023-08-25

**Authors:** Hirokazu Toyoshima, Motoaki Tanigawa, Chiaki Ishiguro, Hiroyuki Tanaka, Yuki Nakanishi, Shigetoshi Sakabe

**Affiliations:** a Department of Infectious Diseases, Japanese Red Cross Ise Hospital, Mie, Japan; b Infection Prevention and Control Office, Japanese Red Cross Ise Hospital, Mie, Japan; c Department of Respiratory Medicine, Japanese Red Cross Ise Hospital, Mie, Japan; d Department of Medical Technology, Japanese Red Cross Ise Hospital, Mie, Japan.

**Keywords:** atlantoaxial subluxation, beta-hemolysis, Lancefield group A, optimal management, pyrrolidonyl arylamidase test, *Streptococcus dysgalactiae* subspecies *equisimilis*, vertebral osteomyelitis

## Abstract

**Rationale:**

Clinically, vertebral osteomyelitis commonly occurs in immunocompromised individuals, such as people with diabetes, immunosuppression, chronic liver disease, and malignancy. Microbiologically, vertebral osteomyelitis is commonly caused by *Staphylococcus aureus*; however, *Streptococcus dysgalactiae* subspecies *equisimilis* (SDSE) may also potentially cause vertebral osteomyelitis, albeit rarely. Since no case reports have documented the occurrence of SDSE cervical osteomyelitis accompanied by progressive atlantoaxial subluxation, its clinical characteristics remain uncertain. Herein, we report the first case of progressive atlantoaxial subluxation in addition to cervical osteomyelitis due to septic atlantoaxial arthritis caused by SDSE in an immunocompetent individual, and provide a review of the relevant literature.

**Patient concerns:**

A 63-year-old man with hypertension but no history of trauma or musculoskeletal disorders presented with worsening neck pain for 1 month without fever. Physical examination revealed neck pain due to neck retroflexion and tenderness with swelling of the upper cervical spine. No neurological deficit was observed. Magnetic resonance imaging revealed low-intensity areas on a T1-weighted image and high-intensity areas on a short tau inversion recovery image at the C2, C5, and C6 vertebral bodies with atlantoaxial subluxation. Two sets of blood culture tests (aerobic and anaerobic) were performed.

**Diagnoses:**

The anaerobic blood culture bottle showed the presence of beta-hemolytic pyrrolidonyl arylamidase-negative SDSE expressing Lancefield group A antiserum. Hence, the patient was diagnosed with SDSE cervical osteomyelitis with atlantoaxial subluxation; intensive intravenous ampicillin (2 g every 6 hours) – which is effective against SDSE – was administered.

**Interventions:**

Posterior fusion (occipital bone, C4) was performed on day 33 because a follow-up magnetic resonance imaging on day 31 revealed progression of atlantoaxial subluxation with thickened atlantodental soft tissue.

**Outcomes:**

The patient’s neck pain was completely relieved after treatment with intravenous ampicillin for 6 weeks, followed by oral amoxicillin (1500 mg) daily for an additional 4 weeks. The patient did not experience recurrence or sequelae during the 2-year follow-up period.

**Lessons:**

SDSE expressing Lancefield group A antiserum can cause afebrile vertebral osteomyelitis and progressive atlantoaxial subluxation due to the occurrence of septic atlantoaxial arthritis in immunocompetent individuals. Spinal instrumentation for vertebral osteomyelitis may be acceptable after 6 weeks of antimicrobial therapy.

## 1. Introduction

Vertebral osteomyelitis commonly involves a hematogenous infection affecting the vertebral body endplates and intervertebral discs.^[1]^ The risk of vertebral osteomyelitis increases with age, and the annual incidence in developed countries is approximately 0.4 to 2.4/100,000.^[1]^ Other predisposing risk factors include diabetes, immunosuppression, chronic liver disease, malignancy, end-stage renal disease, endocarditis, preceding surgical procedures, and intravenous drug abuse.^[2]^ The most common causative microorganism is *Staphylococcus aureus*, whereas vertebral osteomyelitis caused by *Streptococcus dysgalactiae* subspecies *equisimilis* (SDSE) is extremely rare.^[1]^ Vertebral osteomyelitis mainly occurs at the lumbar level (43.1%) and sporadically at the cervical level (10.6%).^[3]^ Infection-related atlantoaxial subluxation, such as Grisel syndrome, is a result of inflammatory ligamentous laxity due to infections, followed by atlantoaxial subluxation.^[4]^ However, the diagnostic and therapeutic strategies for SDSE cervical osteomyelitis with progressive atlantoaxial subluxation remain unclear. Herein, we report a unique case of Lancefield group A SDSE vertebral osteomyelitis with progressive atlantoaxial subluxation and a review of the relevant literature.

## 2. Case presentation

A 63-year-old Japanese man presented to the hospital with complaints of worsening neck pain over a period of 1 month. The patient had hypertension, but no history of trauma or musculoskeletal disorders.

**Figure 1.**
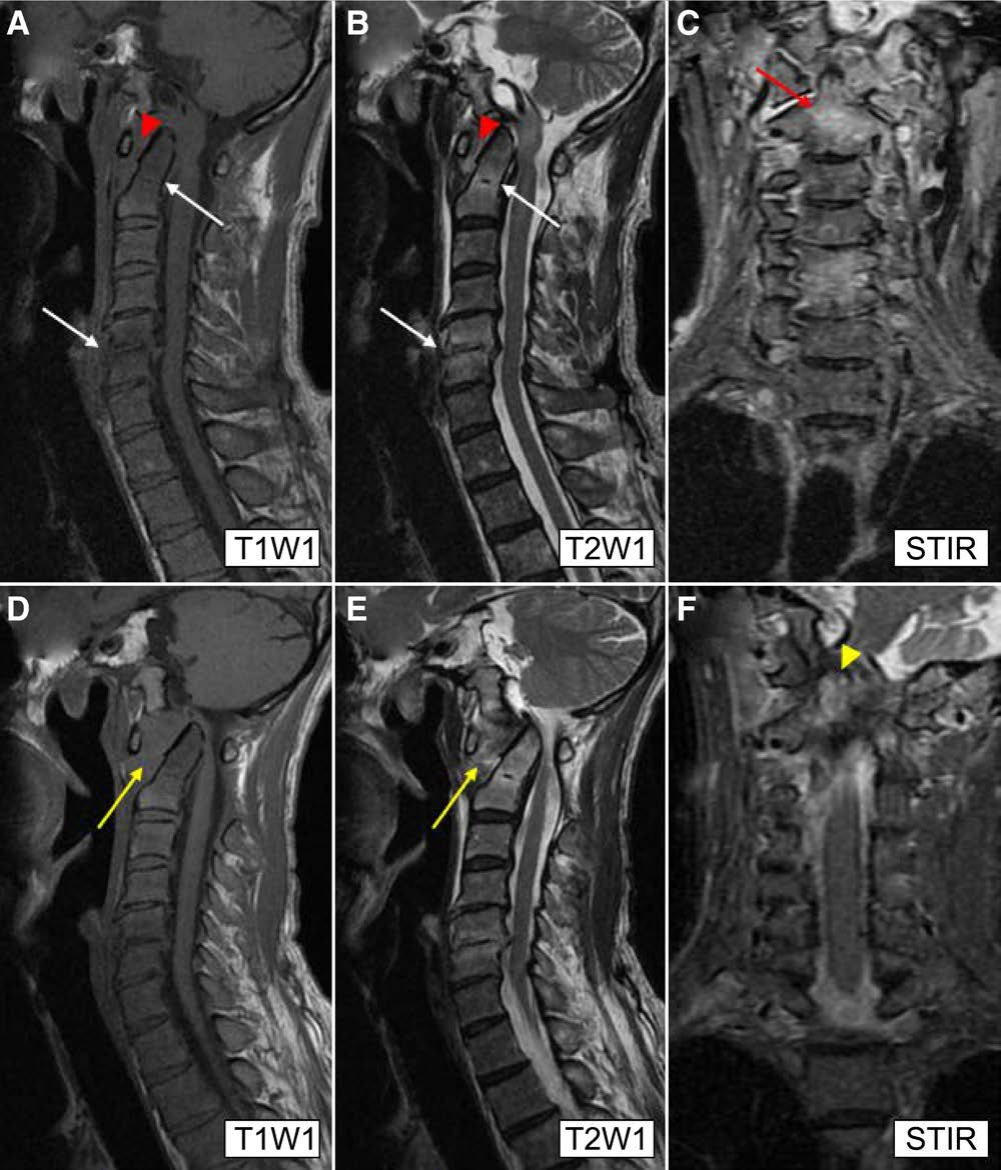
Radiological findings. (A–C) MRI on admission shows a lesion with low T1 (A) and high T2 intensity (B) at the C2, C5, and C6 vertebral bodies and the C5–C6 intervertebral disc in the sagittal section (white arrows). The coronal section (C) shows high-intensity with STIR at the C2 vertebral body (red arrow) in the coronal section. Additionally, atlantoaxial subluxation is suspected with the atlantodental interval of 5.27 mm (red arrowheads) (A, B). (D–F) The follow-up MRI of the sagittal section performed on day 31 reveals exacerbation of the atlantoaxial subluxation with an atlantodental interval of 10.02 mm (yellow arrows) (D, E). Imaging of the coronal section (F) reveals the expansion of a high-intensity area with STIR to the dens (yellow arrowhead). MRI = magnetic resonance imaging, STIR = short tau inversion recovery, T1WI = T1-weighted image, T2WI = T2-weighted image.

The patient was alert and had the following vital signs: body temperature, 36.8°C; blood pressure, 137/92 mm Hg; heart rate, 77 beats/minutes; respiratory rate, 18 breaths/minutes; and oxygen saturation, 99% on ambient air. Physical examination revealed neck pain due to neck retroflexion and tenderness with swelling of the upper cervical spine. No neurological abnormalities were observed. The laboratory findings were as follows: C-reactive protein (CRP) level, 2.4 mg/L (reference range, 0.0–1.0 mg/L); erythrocyte sedimentation rate, 81 mm/hours (reference range, 2–10 mm/hours); and white blood cell count, 4500/µL (reference range, 3800–9000/μL). Magnetic resonance imaging (MRI) revealed atlantoaxial subluxation: a lesion with low T1 and high T2 intensity at the C2, C5, and C6 vertebral bodies and the C5 to C6 intervertebral discs (Fig. 1A and B), and high-intensity with short tau inversion recovery (STIR) at the C2, C5, and C6 vertebral bodies (Fig. 1C).

**Figure 2.**
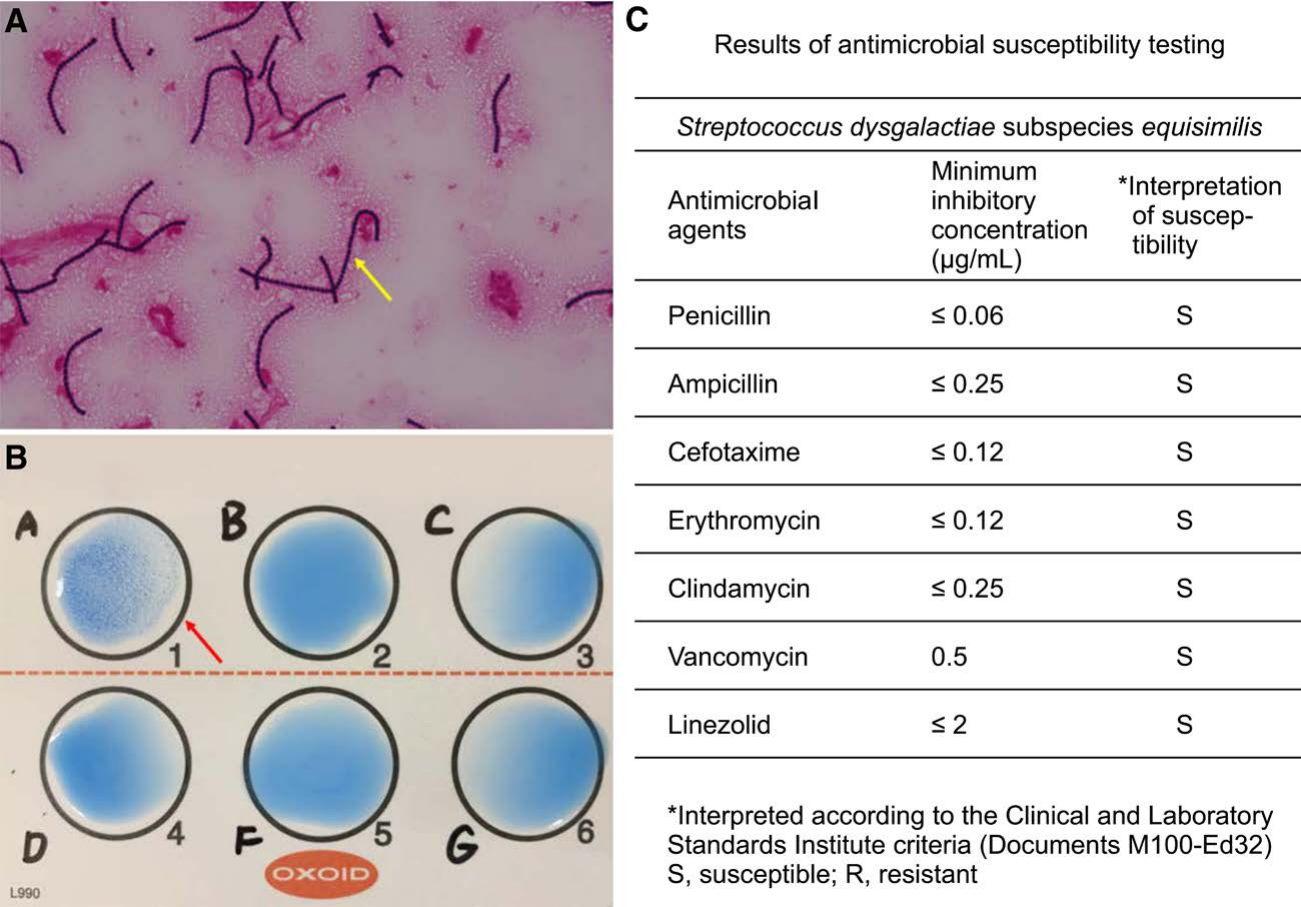
Microbiological findings. (A) Gram staining (×1000) of an anaerobic blood culture bottle reveals gram-positive cocci arranged in long chains (yellow arrow). (B) The isolates show the expression of Lancefield group A antiserum (red arrow). (C) The isolates, identified as SDSE, are susceptible to penicillin. SDSE = *Streptococcus dysgalactiae* subspecies *equisimilis.*

Two sets of blood culture tests (aerobic and anaerobic) were performed for C2, C5, and C6 vertebral osteomyelitis and C5 to C6 discitis. Anaerobic blood culture bottles incubated in the BacT/Alert system (bioMérieux, Marcy I’Etoile, France) for 13 hours showed positive results. Gram staining revealed Gram-positive cocci arranged in long chains (Fig. 2A). Other anaerobic and aerobic blood cultures showed negative test results. The isolates were cultured on 5% sheep blood agar (Nihon Becton–Dickinson, Tokyo, Japan); the results showed beta-hemolysis, absence of catalase, sensitivity to bacitracin, and absence of pyrrolidonyl arylamidase (PYR) with presence of Lancefield group A antiserum (Kanto Chemical Co., Inc., Tokyo, Japan) (Fig. 2B). The *Streptococcus dysgalactiae* pathogen was identified using the MALDI Biotyper software (Bruker Daltonics GmbH, Bremen, Germany) with an identification score of 1.97. The results of the rapid ID 32 Strep system (bioMérieux, Marcy I’Etoile, France) showed that the above pathogen had a 99.8% similarity to SDSE. The isolates were identified as SDSE using the VITEK 2 system (bioMérieux, Marcy l’ Etoile, France) and showed susceptibility to penicillin (Fig. 2C).

The patient was diagnosed with SDSE cervical osteomyelitis accompanied by atlantoaxial subluxation. Transthoracic echocardiography showed no infective endocarditis. The patient was treated with intravenous ampicillin (2 g) every 6 hours, and a follow-up blood culture test carried out on day 7 showed negative results. The patient’s neck pain gradually improved, without limb numbness or palsy. However, follow-up MRI performed on day 31 revealed progression of the atlantoaxial subluxation with thickened atlantodental soft tissue (Fig. 1D and E) and expansion of a high-intensity area with STIR to the dens (Fig. 1F).

**Figure 3.**
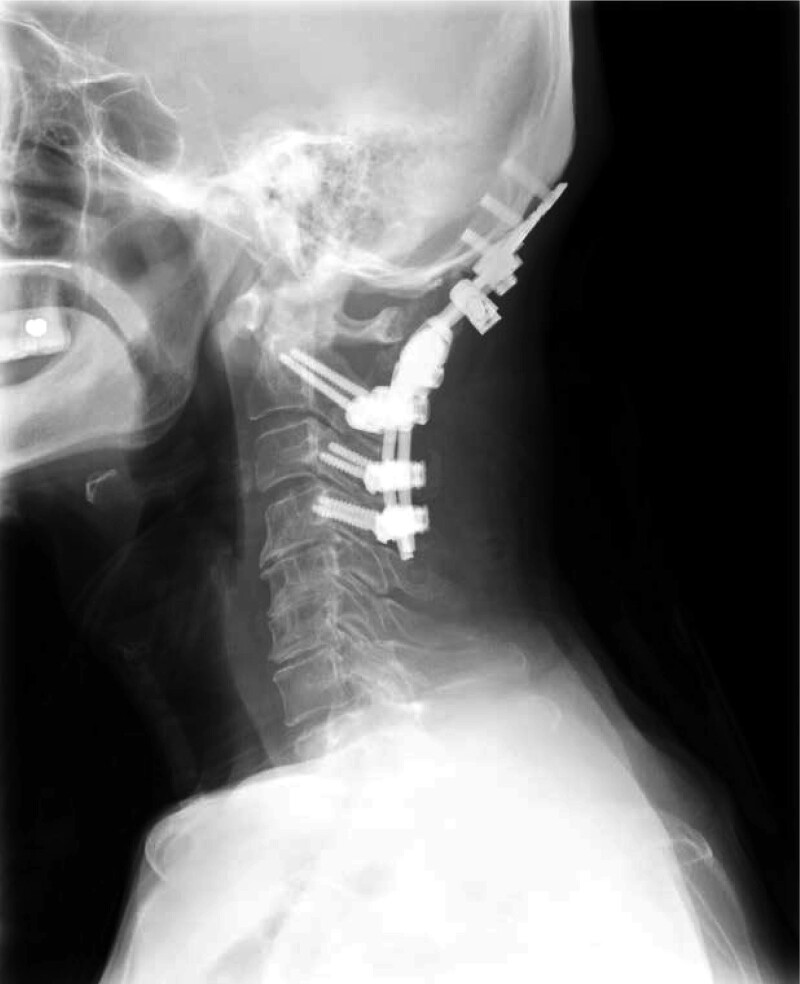
Postoperative radiology. Radiograph at 2 weeks postoperatively shows that the implants are stable without loosening.

Consequently, a posterior fusion procedure (occipital bone, C4) was performed on day 33, considering the involvement of SDSE in the progression of atlantoaxial subluxation. Ampicillin (2 g) was administered intravenously every 6 hours for 6 weeks, followed by oral amoxicillin (1500 mg) daily for an additional 4 weeks. A follow-up radiographic examination performed 2 weeks after surgery showed that the implants were stable without loosening (Fig. 3). The neck pain was relieved, and the patient remained disease free with no recurrence or sequelae during the 2-year follow-up period.

## 3. Discussion and conclusion

This case illustrates 3 clinical issues: The possibility of developing afebrile, normoleukocytic vertebral osteomyelitis caused by SDSE expressing Lancefield group A antiserum in immunocompetent individuals; The relationship between the progression of atlantoaxial subluxation and SDSE infection; and The optimal management of SDSE vertebral osteomyelitis.

Seven English-language case reports (9 cases) have documented the incidence of SDSE vertebral osteomyelitis^[5–11]^ (Table 1). The median age of the patients was 57 years (range, 43–74 years), all of whom were male. Three (33.3%) of the 9 patients developed cervical spine osteomyelitis. This indicates that SDSE more commonly affects the cervical spine compared with other microorganisms (SDSE vs overall, 33.3% vs 10.6%).^[3]^ Eight (88.9%) of the 9 patients had a fever, while 7 (87.5%) of the 8 patients showed leukocytosis (>10,000/μL) and elevated CRP level (>100 mg/L). By contrast, the patient in our case was afebrile with a normal White blood cell count and slightly elevated CRP levels despite the elevated erythrocyte sedimentation rate shown on laboratory testing. All patients who underwent blood culture testing and showed Lancefield group strains were positive for SDSE expressing Lancefield group G antiserum. None of the patients developed Lancefield group A SDSE vertebral osteomyelitis. Four (44.4%) of the 9 patients had skin and soft tissue infections, while 1 (11.1%) had a catheter-related bloodstream infection as the primary source of infection. Our patient did not have the predisposing risk factors for vertebral osteomyelitis,^[2]^ and there was no evidence of the related source, such as pharyngitis, skin and soft tissue infection, and wound or abrasion on physical examination. To the best of our knowledge, this is the first case report to document the incidence of Lancefield A SDSE vertebral osteomyelitis with apyrexia and approximately normal acute-phase reactants without an apparent primary source. Generally, Lancefield A gram-positive *Streptococcus* arranged in long chains resembles *S. pyogenes*; however, SDSE – which is assumed to be Lancefield group C or G *Streptococcus* resembling *S. pyogenes* in Gram staining – rarely expresses Lancefield group A antiserum.^[12]^ Moreover, Lancefield group A SDSE causes invasive infection, such as severe skin soft tissue infection, pneumonia, and septic shock.^[12]^ To distinguish these microorganisms – even in a clinical setting – the PYR test is helpful as it can specifically distinguish *S. pyogenes* from other beta-hemolytic streptococcal organisms, despite having the same catalase-negative and bacitracin-sensitive features.

**Table 1:** Vertebral osteomyelitis caused by *Streptococcus dysgalactiae* subspecies *equisimilis*.

Case	Age (yr)	Sex	Predisposing factors	Sites	Clinical features	Laboratory data	Samples	Blood cultures	Lancefield grouping	Infections in other sites	Primary source of infection	Duration of antimicrobial treatment (wk)	Outcome	Sequelae
Case 1 ^[5]^	52	M	Chemotherapy and irradiation therapy for nasopharyngeal carcinoma	C6–C7	Posterior neck and upper arm pain Fever	WBC: 13,400/µL CRP: 270.8 mg/L ESR: NS	Blood culture	Positive	NS	None	CRBSI	16	A	None
Case 2 ^[6]^	63	M	None	T4–S1	Right hand and lower back pain Fever	WBC: 34,100/µL CRP: 365 mg/L ESR: NS	Blood culture	Positive	NS	Septic arthritis	Cellulitis Intramuscular injection	10	A	None
Case 3 ^[7]^	57	M	Alcohol abuse Dental care Right knee replacement	L2–L3	Hemiparesis Dysarthria Confusion	WBC: 19,850/µL CRP: 333 mg/L ESR: NS	Blood culture Right knee purulent material culture	Positive	NS	Meningitis Disseminating foci in the joints and soft tissue	Cellulitis with thrombosis	12	A	None
Case 4 ^[8]^	74	M	Diabetes mellitus	C2–C3 L2–L3	Lower back and neck pain Fever	WBC: 9970/µL CRP: 276.7 mg/L ESR: 105 mm/h	Blood culture	Positive	NS	None	Cellulitis	10	A	None
Case 5 ^[9]^	59	M	Coronary heart disease Myelodysplastic syndrome Chronic recurrent pancreatitis Seminoma	L4–L5	Lower back and neck pain Fever	WBC: 12,400/µL CRP: 241 mg/L ESR: 105 mm/h	Blood culture swab material from a leg ulcer	Positive	G	None	Leg ulcer	8	A	None
Case 6 ^[10]^	57	M	None	C4–C5 L3–L4	Lower back and neck pain Fever Hand swelling Bloody stool	WBC: 15,400/µL CRP: 210 mg/L ESR: NS	Blood culture	Positive	G	Reactive arthritis	Intestinal amebiasis	28	A	None
Case 7 ^[11]^	55	M	None	L1–L2	Back pain Fever	WBC: 15,100/µL CRP: NA ESR: NS	Needle biopsy culture	NA	NS	None	Arthritis	12	A	None
Case 8 ^[11]^	43	M	None	L5–S1	Back pain Confusion Lower limb weakness Fever	WBC: 15,800/µL CRP: 347 mg/L ESR: NS	Blood culture	Positive	NS	Infective endocarditis	None	6	A	None
Case 9 ^[11]^	58	M	None	T11–T12	Back pain Fever	WBC: NS CRP: 50 mg/L ESR: 63 mm/h	Blood culture Needle biopsy culture	Positive	NS	None	None	6	A	None

A = alive, CRBSI = catheter-related blood stream infection, CRP = C-reactive protein, ESR = erythrocyte sedimentation rate, M = male, NA = not applicable, NS = not stated, WBC = white blood cell.

In our case, the patient had cervical osteomyelitis at the C2, C5, and C6 vertebral bodies and the C5 to C6 intervertebral disc as well as progressive atlantoaxial subluxation. MRI performed on admission with STIR revealed high-intensity at the C2 vertebral body, which expanded to the dens as revealed on follow-up MRI (Fig. 1C and F). The patient had no previous history of tonsillectomy and adenoidectomy, crown dens syndrome, or rheumatoid arthritis. This case indicates that infection with SDSE can cause septic atlantoaxial arthritis in addition to cervical osteomyelitis. Septic atlantoaxial arthritis is an extremely rare condition; however, *S. aureus* is the most common causative microorganism.^[13]^ No previous case reports have documented the occurrence of septic atlantoaxial arthritis caused by SDSE (Table 1). Clinicians should consider SDSE in addition to *S. aureus* as a causative microorganism of septic atlantoaxial arthritis.

The optimal antimicrobial therapy duration for vertebral osteomyelitis in patients with SDSE remains unclear. McHenry MC et al^[3]^ reported that at least 4 to 6 weeks of specific intravenous antimicrobial treatment, followed by additional appropriate oral antimicrobial therapy, is required to achieve favorable outcomes in patients with vertebral osteomyelitis. By contrast, Bernard L et al^[14]^ reported that 6 weeks of specific antimicrobial treatment was not inferior to 12 weeks of specific antimicrobial treatment for vertebral osteomyelitis. Additionally, both groups were only treated with intravenous antimicrobials for 2 weeks (median).^[14]^ Park KH et al^[15]^ reported that spinal instrumentation was not associated with the recurrence of vertebral osteomyelitis, and the recurrence was correlated with the duration of antimicrobial therapy. In this report, the recurrence was more common in patients with < 6 weeks of antimicrobial therapy than in those with ≥ 6 weeks of antimicrobial therapy.^[15]^ In previous case reports of SDSE vertebral osteomyelitis, patients received antimicrobial therapy for 10 weeks (median) (Table 1). In our case, parenteral ampicillin was administered for 6 weeks, followed by oral amoxicillin for an additional 4 weeks, owing to the necessity of performing spinal instrumentation for spinal fixation. Concerning the optimal timing of this procedure, Park et al^[15]^ reported that spinal instrumentation should not be delayed as the clinical outcomes in patients with spinal instrumentation for vertebral osteomyelitis were similar to those with spinal non-instrumentation. Although the patient underwent posterior fusion (occipital bone, C4) on day 33 to manage the progressive atlantoaxial subluxation despite receiving appropriate antimicrobial therapy^[16]^ and eventually recovered without experiencing recurrence and sequelae, the optimal timing for surgical intervention warrants further evaluation in the future by conducting additional case reports of septic atlantoaxial arthritis caused by SDSE.

A limitation of this case is that the optimal timing of follow-up MRI for vertebral osteomyelitis accompanied by progressive atlantoaxial subluxation caused by SDSE remains unknown. Prompt surgical intervention based on early follow-up MRI findings might have shortened the hospitalization period.

In conclusion, SDSE expressing Lancefield Group A antiserum can cause afebrile normoleukocytic vertebral osteomyelitis and progressive atlantoaxial subluxation due to the occurrence of septic atlantoaxial arthritis in immunocompetent individuals. Lancefield group A SDSE could be distinguished from *S. pyogenes* using the PYR test. Spinal instrumentation for vertebral osteomyelitis may be acceptable after 6 weeks of antimicrobial therapy.

## Acknowledgments

We would like to thank Editage (http://www.editage.com) for editing and reviewing this manuscript.

## Author contributions

**Conceptualization:** Hirokazu Toyoshima, Chiaki Ishiguro.

**Data curation:** Hirokazu Toyoshima, Chiaki Ishiguro.

**Methodology:** Hirokazu Toyoshima, Chiaki Ishiguro, Hiroyuki Tanaka, Yuki Nakanishi.

**Supervision:** Motoaki Tanigawa, Shigetoshi Sakabe.

**Visualization:** Hirokazu Toyoshima.

**Writing – original draft:** Hirokazu Toyoshima.

**Writing – review & editing:** Hirokazu Toyoshima.
